# Co-infections observed in SARS-CoV-2 positive patients using a rapid diagnostic test

**DOI:** 10.1038/s41598-021-95772-3

**Published:** 2021-08-11

**Authors:** Carla Fontana, Marco Favaro, Silvia Minelli, Maria Cristina Bossa, Anna Altieri

**Affiliations:** 1 Department of Experimental Medicine, University of Rome Tor Vergata, Via Montpellier 1, 00133 Rome, Italy; 2 Microbiology and Virology Lab, Tor Vergata University Hospital, V.le Oxford, 81 00133 Rome, Italy

**Keywords:** Clinical microbiology, Molecular medicine

## Abstract

Rapid diagnostic tests are tools of paramount impact both for improving patient care and in antimicrobial management programs. Particularly in the case of respiratory infections, it is of great importance to quickly confirm/exclude the involvement of pathogens, be they bacteria or viruses, while obtaining information about the presence/absence of a genetic target of resistance to modulate antibiotic therapy. In this paper, we present our experiences with the use of the Biofire® FilmArray® Pneumonia Panel Plus (FAPP; bioMérieux; Marcy l’Etoile, France) to assess coinfection in COVID-19 patients. A total of 152 respiratory samples from consecutive patients were examined, and 93 (61%) were found to be FAPP positive, with the detection of bacteria and/or viruses. The patients were 93 males and 59 females with an average age of 65 years who were admitted to our hospital due to moderate/severe acute respiratory symptoms. Among the positive samples were 52 from sputum (SPU) and 41 from bronchoalveolar lavage (BAL). The most representative species was *S. aureus* (most isolates were *mec*A positive; 30/44, 62%), followed by gram-negative pathogens such as *P. aeruginosa*, *K. pneumoniae*, and *A. baumannii*. Evidence of a virus was rare. Cultures performed from BAL and SPU samples gave poor results. Most of the discrepant negative cultures were those in which FAPP detected pathogens with a microbial count ≤ 10^5^ CFU/mL. *H. influenzae* was one of the most common pathogens lost by the conventional method. Despite the potential limitations of FAPP, which detects a defined number of pathogens, its advantages of rapid detection combined with predictive information regarding the antimicrobial resistance of pathogens through the detection of some relevant markers of resistance could be very useful for establishing empirical targeted therapy for the treatment of patients with respiratory failure. In the COVID era, we understand the importance of using antibiotics wisely to curb the phenomenon of antibiotic resistance.

Since November 2019, the world has faced an unprecedented public health emergency caused by the coronavirus named SARS-CoV-2 and the disease known as COVID-19^1–4^. The pandemic has put pressure on the entire health system. Most importantly, microbiologists have faced serious diagnostic difficulties and have had to respond to medical needs without reliable scientific evidence, especially in the early stages of the pandemic^5^. One fascinating aspect that was underestimated at the start of the pandemic was the role that bacterial and fungal infections played in the evolution of COVID-19. Considering the evidence that the symptoms of COVID-19 are highly similar to those of other respiratory infections, differential diagnosis is the most important requirement^6^. Therefore, in the diagnostic algorithm for pneumonia, we must first determine whether we are facing a viral infection, and in this case, whether it is SARS-CoV-2 or another viral agent. However, it is also important during the diagnostic process to confirm or exclude the possibility of overinfection caused by bacteria and/or fungi that may also affect mortality^7,8^. In different reports, the incidence of coinfection varies; for example, Ruan et al. reported that in Wuhan, 15% of surviving hospitalized patients and up to 50% of nonsurvivors had coinfection^9^. Recently, Lansbury et al., in a systematic review, reported that 7% of hospitalized COVID-19 patients experienced coinfection, and this proportion doubled among patients in the ICU^10^. It has also been reported that the empirical use of antibiotics in most COVID-19 patients may be related to bacterial infection/overinfection^11–13^. The NICE COVID-19 guidelines also recommend using antibacterial agents wisely^14^. In particular, microbiological evidence indicates that pathogens such as *Streptococcus pneumoniae*, *Hemophilus influenzae*, *Staphylococcus aureus*, and *Aspergillus* spp. are often associated with a poor prognosis in COVID-19 patients^15–19^.

However, as reported by Giacobbe et al., overinfection and/or coinfection of the respiratory tract is not the only worrisome aspect of COVID-19. The incidence of bloodstream infection (BSI) is also high^20^. Rapid identification of coinfection can help save lives^8^. For such purposes, a rapid diagnostic test (RDT) may be the right choice, especially when processing respiratory samples. Recently, Calcagno et al. demonstrated the usefulness of the Biofire® FilmArray® Pneumonia Panel (BioFire Diagnostics, bioMerieux) for assessing coinfection in COVID-19 patients^21^. In this article, we introduced our experience with using the Biofire® FilmArray® Pneumonia Panel Plus system (FAPP; the new version of the Biofire® FilmArray® Pneumonia Panel) to identify SARS-CoV-2-positive patients with possible coinfections. This paper does not aim to compare the FAPP with traditional culture methods and analyze the differences; instead, it aims to emphasize the usefulness of RDT for quickly ruling out coinfections or superinfections to treat them correctly and avoid the unnecessary use of antibiotics. As Rawson et al. showed in their comments on hospitalized COVID-19 patients, the latter problem has a large impact. In fact, his research shows that although only 8% of the evidence shows the superimposition of bacterial or fungal coinfections, approximately 72% of patients receive antibiotic treatment^22^.

## Materials and methods

### Specimens

This study included 152 respiratory specimens (66 from bronchoalveolar lavage fluid and 86 from sputum) collected from patients admitted to Tor Vergata University Hospital due to respiratory failure between March 2020 and June 2020. They were 93 males and 59 females (average age 65 years) who were admitted to our hospital due to moderate/severe acute respiratory syndrome, and 23 of them required assisted ventilation in the ICU (10 females and 13 males with average ages of 73.4 and 75,5, respectively). Due to the COVID-19 pandemic, nasopharyngeal swabs were also collected from all patients for SARS-CoV-2 assay. The samples were collected by well-trained staff using a nasopharyngeal Eswab™ (Copan, Brescia-Italy) and were analyzed using the Allplex™ 2019-nCov Assay (Seegene).

### Traditional methods for the routine analysis of respiratory tract specimens

Respiratory samples from bronchoalveolar lavage fluid (BAL) and sputum (SPU) were Gram-stained and cultured according to standard protocols to detect the most common respiratory pathogens^23^. Sputasol buffer solution (Thermo Fisher, Gloucester-UK) was used to fluidize the mucus sample. Sputasol was prepared according to the manufacturer’s instructions. Part of the SPU or BAL secretion sample was diluted 1:1 (v/v) in this buffer, vortexed to mix, and shaken for 15 min at room temperature. Ten microliters of the treated sample was inoculated on five different types of agar plates: two plates each of Columbia CNA (one incubated under anaerobic conditions), MacConkey agar, Burkholderia selective agar, chocolate agar, and Saboraud dextrose agar (bioMérieux).

The BAL fluid was centrifuged at 4000 g for 20 min in advance, and then an aliquot of the pellet was streaked onto the surface of the same set of agar plate media. The culture was incubated at 37 °C for 24 h under aerobic, microaerobic and anaerobic conditions (Columbia CNA and chocolate agar plates). At the end of the incubation, the plates were examined for the presence of significant pathogens; if they were negative, they were kept for further observation for up to 5 days. Gram smears were collected using ten µL of the pellet, air dried, fixed at 42 °C, and stained using PREVI COLOR (bioMérieux). Validation was carried out according to the Murray Washington scheme. Microbial counts (expressed in CFU/ml) were performed using the HB&L system (Alifax, Padova, Italy)^24^.

The MALDI TOF MS system (Bruker Daltonics, Bremen, Germany) was used to identify significant pathogens (those shown in Tables 3 and 4), the Vitek 2 system (bioMerieux) and the Micronaut panel (Diagnostika Gmbh, Bornheim, Germany, now Bruker Daltonics) running on MICRO MIB (Bruker Daltonics) were used for antimicrobial susceptibility testing. Micronaut panels (MIBs) were used to confirm the resistance phenotypes detected using the Vitek 2 system. MIB allows the determination of the MIC using the broth microdilution method, as recommended by the EUCAST standard. The ASTs were interpreted according to EUCAST clinical breakpoints v 10.0^25^.

### Blood cultures

Blood samples were collected in BacT/ALERT FN Plus and BacT/ALERT FA Plus vials (bioMérieux; Marcy l’Etoile, France) and incubated for up to 5 days in the Virtuo system or until they signaled *positive* or otherwise negative.

### Biofire®FilmArray® Pneumonia plus (FAPP)

FAPP is a syndromic panel based on multiplex PCR. targeting 18 bacterial pathogens (11 g-negative, 4 g-positive and 3 atypical), 9 viruses and 7 determinants of resistance (namely, CTX-M, KPC, NDM, Oxa48-like, VIM, IMP, mecA/mecC and MREJ). Viral, fungal and atypical bacterial detections were reported as not detected or detected, and resistance genes were reported as positive. In the case of positive results, semiquantitative values expressed in DNA copies/ml are also reported for each pathogen detected.

All steps from nucleic acid extraction to the final detection of pathogens were carried out in an automated manner. The sample swab included in the kit was used to dispense the appropriate amount of SPU and/or BAL into the cartridge according to the manufacturer’s instructions. Briefly, approximately 200 μL of the sample was collected using a flocked swab and transferred to a sample injection vial. It was then mixed with the provided sample buffer. This solution was then loaded into the FilmArray pouch, which in turn was loaded into the FilmArray platform. The preparation of each cartridge require approx. 2 min of hands-on time, while the run time was approximately one hour and 15 min. The principle and procedure of the assay have already been described^26,27^.

All methods described were carried out in accordance with relevant guidelines and regulations.

### Ethics approval

According to the CODE guideline ethics approval is not necessary for retrospective studies in which all data analysed were collected as part of routine diagnosis. Specific informed consent was waived due to the retrospective nature of the study: “*Comitato Etico Indipendente”* (D.M. 8 febbraio 2013) Fondazione Policlinico Tor Vergata also approved experimental protocol (prot 102/20).

## Results

The study included 152 respiratory samples from different consecutive patients. All patients were positive for SARS-CoV-2. Additionally, FAPP-positive bacteria and/or viruses were present in 93 (52 SPU and 41 BALs; 61%) patients. The positive samples comprised 52 SPU and 41 BAL samples (see Tables 1 and 2).

**Table 1:** Microorganism detected by Biofire®FilmArray® Pneumonia plus in BAL.

No of samples	Microrganism detected	Target of resistance	CFU/mL
8	*S.aureus*	mec A	10^4^–10^7^
2	*S.aureus*		10^4^–10^7^
1	*S.aureus*, *H.influenzae*, Influenza A	mec A	10^5^–10^7^
1	*S.aureus,* Influenza A + B	mec A	10^7^
2	*S.aureus, P.aeruginosa*	mec A	10^4^–10^7^
1	*S.aureus, P.aeruginosa*, Human Metapneumovirus	VIM	10^5^–10^7^
1	*S.aureus, E.coli, H.influenzae*		10^7^
2	*S.aureus, K.pneumoniae*	mec A, CTX-M	10^4^–10^6^
1	*S.aureus, K.pneumoniae*	KPC	10^4^
1	*S.aureus, K.oxytoca*	mec A	10^4^
1	*A.baumannii/calcoaceticus, K.pneumoniae, P.aeruginosa,* Coronavirus		10^4^
3	*A.baumannii/calcoaceticus*		10^5^–10^7^
1	*A.baumannii/calcoaceticus, P.aeruginosa*		10^5^–10^7^
1	*S.pneumoniae*		10^4^
1	*S.pneumoniae, H.influenzae, M.catharralis*		10^4^
2	*E.coli*		10^4^
1	*E.cloacae,K.oxytoca, E.coli*		10^7^
2	*K.pneumoniae*	CTX-M	10^4^–10^5^
1	*S.aureus, K.pneumoniae, P.aeruginosa*	mecA, KPC, VIM	10^5^–10^7^
1	*K.pneumoniae*	KPC	10^5^
1	*K.pneumoniae, Proteus spp, E.cloacae*	CTX- M IMP	10^4^–10^5^
2	*P.aeruginosa*	VIM	10^5^–10^7^
1	*P.aeruginosa*		10^6^
1	*E.cloacae*		10^7^
1	*E.cloacae*	VIM	10^4^
1	Influenza A

**Table 2:** Microorganism detected by Biofire®FilmArray® Pneumonia plus in Sputum.

No. of samples	Microroganism detected	Target of resistance	CFU/mL
1	*P.aeruginosa*		10^5^
1	*K.oxytoca, H.influenzae*		10^4^–10^5^
1	*S.aureus, S.pneumoniae, H.influenzae*	mec A	10^4^–10^6^
1	*E.cloacae, K.pneumoniae, P.aeruginosa, S.aureus*		10^4^–10^7^
1	*H.influenzae*		10^6^
1	*E.coli, M.catarrhalis*		10^4^–10^5^
1	*S.aureus, H.influenzae*		10^4^–10^5^
1	*M.catarrhalis*		10^5^
1	*S.aureus, Human metapneumovirus*		10^4^
1	*E.coli, P.aeruginosa, H.influenzae*	VIM	10^4^–10^5^
2	*P.aeruginosa*		106
1	*S.aureus, H.influenzae*	mecA	10^4^–10^7^
1	*E.coli, S.aureus, E.cloacae, K.pneumoniae*	CTX-M	10^4^–10^6^
1	*M.catharralis*		10^4^
1	*E.cloacae, K.pneumoniae, P.aeruginosa, S.aureus*		10^4^–10^7^
1	*H.influenzae, M.catarrhalis, P.aeruginosa*		10^7^
1	*E.cloacae, Koxytoca*	KPC	10^4^–10^7^
1	*A.baumannii/calcoaceticus*		10^4^
1	*A.baumannii/calcoaceticus, E.coli*		10^4^–10^7^
1	*E.coli*		10^7^
1	*A.baumannii/calcoaceticus, E.coli, K.pneumoniae*	CTX-M, KPC	10^7^
1	*S.marcescens*		10^4^
1	*E.coli, S.aureus*	CTX-M	10^4^–10^7^
1	*M.catharralis, E.cloacae, K.oxytoca*		10^4^–10^5^
1	*K.pneumoniae, S.aureus*	mec A	10^6^–10^7^
1	*K.pneumoniae*	CTX-M	10_6
1	*P.aeruginosa, S.aureus*		10^4^–10^5^
1	*H.influenzae*		10^5^
1	*S.aureus, Adenovirus*		10^7^
1	*K.aerogenes*		10^7^
1	*P.aeruginosa, S.aureus*	mec A	10^6^
1	*P.aeruginosa, S.marcescens*		10^5^
1	*E.cloacae*		10^7^
1	*H.influenzae, M.catharralis, S.pneumoniae*		10^5^–10^6^
2	*P.aeruginosa*		10^6^–10^7^
10	*S.aureus*	mec A	10^4^–10^7^
5	*S.aureus*		10^4^–10^7^

The most representative species was *S. aureus* in both BAL (21; 16 mecA positive) and SPU (27; 14 mecA positive), and the majority were *mec*A positive (30/44, 62%). In most cases, *S. aureus* was the cause of coinfections associated with gram-negative bacteria (such as Enterobacterales and nonfermenting gram-negative bacilli). It was also common to detect *H. influenzae* (9 positive SPU samples and 3 positive BAL samples) in combination with Enterobacterales, *P. aeruginosa*, and *Moraxella catarrhalis* or gram-positive bacteria (such as *S. pneumoniae*).

Most of the gram-negative bacteria detected were Enterobacterales, such as *K. pneumoniae*, *Enterobacter cloacae*, *Klebsiella aerogenes*, and *Serratia marcescens*, followed by gram-negative nonfermenting bacilli, such as *P. aeruginosa*, *Acinetobacter baumannii/calcoaceticus, H. influenzae*, and *M. catarrhalis*.

In SPU or BAL, the most frequently established gram-negative pathogens were *P. aeruginosa* (detected in 12 SPU samples and 9 BAL samples), followed by *K. pneumoniae* (in 6 SPU and 9 BAL samples). *S. pneumoniae* was detected in only two samples each of BAL and SPU (four samples total).

Interestingly, in three cases, viral agents such as influenza A/B virus and human metapneumovirus in BAL were also found (two samples were related to bacteria). In SPU, we found only two specimens with viruses, namely, adenovirus and human metapneumovirus combined with *S. aureus.*

Table 3 shows the comparison results for culture and FAPP in SPU. Of the 52 FAPP-positive samples, 34 were culture negative, and 18 were culture positive (15 positive samples were concordant with the FAPP results, while three showed different pathogens). Of the 18 positive samples that were cultured, most of them lost at least one pathogen (among the 2–4 pathogens detected by FAPP), and in six samples, only the following pathogens were proven by culture: *Serratia liquefaciens*, *Enterococcus faecium*, *Enterococcus faecalis* plus *K. pneumoniae*, *E. faecium* plus *K. pneumoniae*, *P. aeruginosa, and K. pneumoniae* (see Table 3). The microbial counts of the cultures were approximately one to two times lower than those recorded by FAPP. In two samples, *Aspergillus fumigatus* and *C. albicans* were observed only by culture.

**Table 3:** Comparative results of culture and Biofire®FilmArray® Pneumonia plus in Sputum.

No. of samples	Microorganism detected by FAPP	Target of resistance	CFU/mL	Culture results with CFU/mL observed*
1	*P.aeruginosa*		10^5^	*P.aeruginosa* 10^5^
1	*K.oxytoca, H.influenzae*		10^4^–10^5^	Negative
1	*S.aureus, S.pneumoniae, H.influenzae*	mec A	10^4^–10^6^	Negative
1	*E.cloacae, K.pneumoniae, P.aeruginosa, S.aureus*		10^4^–10^7^	Negative
1	*H.influenzae*		10^6^	Negative
1	*E.coli, M.catarrhalis*		10^4^–10^5^	Negative
1	*S.aureus, H.influenzae*		10^4^–10^5^	*C.albicans* 10^3^
1	*M.catarrhalis*		10^5^	Negative
1	*S.aureus , Human Metapneumovirus*		10^4^	Negative
1	*E.coli, P.aeruginosa, H.influenzae*	VIM	10^4^–10^5^	*E.coli, P.aeruginosa* 10^4^
2	*P.aeruginosa*		10^4^–10^7^	Negative
1	*S.aureus, H.influenzae*	mecA	10^4^–10^7^	Negative
1	*E.coli, S.aureus, E.cloacae, K.pneumoniae*	CTX-M	10^4^–10^6^	*E.coli, S.liquefaciens* 10^5^
1	*M.catharralis*		10^4^	Negative
1	*E.cloacae, K.pneumoniae, P.aeruginosa, S.aureus*		10^4^–10^7^	*P.aeruginosa, K.pneumoniae* 10^6^
1	*H.influenzae, M.catarrhalis, P.aeruginosa*		10^7^	*P.aeruginosa, A.baumannii* 10^6^, *A.fumigatus* (10^3^)
1	*E.cloacae, Koxytoca*	KPC	10^4^–10^7^	*K.oxytoca* KPC (10^6^)
1	*A.baumanni/calcoaceticus*		10^4^	Negative
1	*A.baumanni/calcoaceticus, Ecoli*		10^4^–10^7^	*A.baumannii*, *E.faecium* 10^6^
1	*E.coli*		10^7^	*E.coli, K.pneumoniae, E.faecalis* (10^5^)
1	*A.baumanni/calcoaceticus, K.pneumoniae*	CTX-M, KPC	10^7^	*A.baumannii, P.aeruginosa, K.pneumoniae* 10^5^
1	*S.marcescens*		10^4^	*S.marcescens, C.utilis* (10^3^)
1	*E.coli, S.aureus*	CTX-M	10^4^–10^7^	Negative
1	*M.catharralis, E.cloacae, K.oxytoca*		10^4^–10^5^	*E.cloacae*, *Koxytoca* 10^4^
1	*K.pneumoniae, S.aureus*	mec A	10^6^–10^7^	*K.pneumoniae* 10^5^
1	*K.pneumoniae*	CTX-M	10_6	Negative
1	*P.aeruginosa, S.aureus*		10^4^–10^5^	*P.aeruginosa* 10^4^
1	*H.influenzae*		10^5^	Negative
1	*S.aureus, Adenovirus*		10^7^	Negative
1	*K.aerogenes*		10^7^	Negative
1	*P.aeruginosa, S.aureus*	mec A	10^6^	Negative
1	*P.aeruginosa, S.marcescens*		10^5^	Negative
1	*E.cloacae*		10^7^	Negative
1	*H.influenzae, M.catharralis, S.pneumoniae*		10^5^–10^6^	Negative
2	*P.aeruginosa*		10^6^–10^7^	(1) *K. pneumoniae* 10^4^; 1 Negative
10	*S.aureus*	mec A	10^4^–10^7^	(2) *S.aureus* 10^7^ (8 Negative)
5	*S.aureus*		10^4^–10^7^	(1) *K.pneumoniae* + *E.faecium* (10^4^); (4 Negative)
34	*Negative*			Negative

*= Identification of significant pathogens was performed using MALDI TOF MS (Bruker Daltonics).

The remaining 34 SPU specimens were negative with both methods.

**Figure 1:**
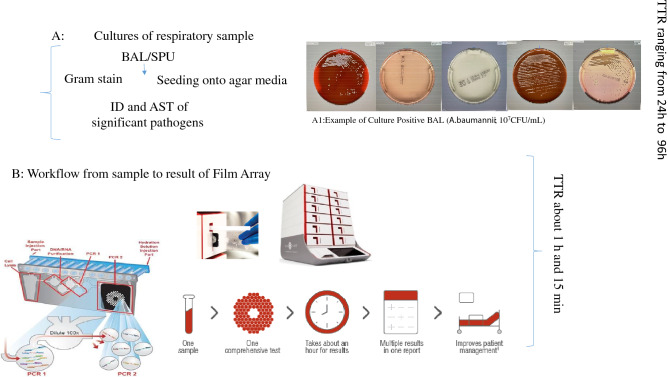
Workflow comparison of cultures and Film Array. Box A: shows the workflow of cultures; Box A1 shows images of *A. baumannii* isolate grown onto solid media; Box B: shows the pathway of FAPP (Images in Box B are gently provided by bioMérieux).

Table 4 shows the comparison results for culturing and FAPP for BAL specimens. For the culture assay, 20 out of 41 FAPP-positive BAL samples were negative, while 21 were culture positive (one BAL sample was only positive for influenza). The negative BAL samples with the largest differences were those for which pathogenic bacteria were detected by FAPP with a microbial count ≤ 10^5^ CFU/mL. In one sample, *E. faecium* was identified by culture but not by FAPP. In contrast, FAPP detected *S. aureus*. In another sample, *S. pettenkoferi* was identified by culture, but not by FAPP (this microorganism is not among the pathogens the molecular assay can identify). Again, as reported above for the SPU samples, fungi was only observed with the culture method. The 25 FAPP-negative BAL samples were also culture negative. Figure 1 reports the comparison of culture and FAPP by sample type and results.

**Table 4:** Comparative results of culture and FAPP in BAL.

No of samples	Microrganism detected by FAPP	Target of resistance	CFU/mL	Culture results with CFU/ml observed*
8	*S.aureus*	mec A	10^4^–10^7^	2 *S.aureus* 10^6^; 6 Negative
2	*S.aureus*		10^4^–10^7^	1 (*E.faecium*); 1 Negative
1	*S.aureus*, *H.influenzae*, Influenza A	mec A	10^5^–10^7^	1 *S.aureus* 10^5^
1	*S.aureus,* Influenza A + B	mec A	10^7^	1 *S.aureus* 10^5^
2	*S.aureus, P.aeruginosa*	mec A	10^4^–10^7^	1 *S.aureus, P.aeruginosa* 10^5^; 1 Negative
1	*S.aureus, P.aeruginosa*, *Human Metapneumovirus*	VIM	10^5^–10^7^	*P.aeruginosa* 10^6^
1	*S.aureus, E.coli, H.influenzae*		10^7^	1 *E.coli, S.aureus* 10^6^
2	*S.aureus, K.pneumoniae*	mec A, CTX-M	10^4^–10^6^	1 *S.aureus, K.pneumoniae* 10^5^; 1 Negative
1	*S.aureus, K.pneumoniae*	KPC	10^6^	*S.aureus, K.pneumoniae* 10^4^
1	*S.aureus, K.oxytoca*	mec A	10^4^	Negative
1	*A.baumanni/calcoaceticus, K.pneumoniae, P.aeruginosa, Coronavirus*		10^4^	Negative
3	*A.baumanni/calcoaceticus*		10^5^–10^7^	2 *A.baumannii* 10^5^; 1 Negative
1	*A.baumanni/calcoaceticus, P.aeruginosa*		10^5^–10^7^	*A.baumannii* 10^5^
1	*S.pneumoniae*		10^4^	Negative
1	*S.pneumoniae, H.influenzae, M.catharralis*		10^4^	Negative
2	*E.coli*		10^4^	2 Negative (1 *C.glabrata*)
1	*E.cloacae,K.oxytoca, E.coli*		10^7^	*E.cloacae,K.oxytoca, E.coli* 10^5^
2	*K.pneumoniae*	CTX-M	10^4^–10^5^	2 *K.pneumoniae* 10^3^
1	*S.aureus, K.pneumoniae, P.aeruginosa*	mecA, KPC, VIM	10^5^–10^7^	1 (*S.aureus, S.pettenkoferi, K.pneumoniae*) 10^6^
1	*K.pneumoniae*	KPC	10^5^	*K.pneumoniae* 10^3^
1	*K.pneumoniae, Proteus spp, E.cloacae*	CTX- M IMP	10^4^–10^5^	Negative
2	*P.aeruginosa*	VIM	10^5^–10^7^	1 *P.aeruginosa* 10^6^ , 1 Negative
1	*P.aeruginosa*		10^6^	Negative
1	*E.cloacae*		10^7^	*E.cloacae* 10^5^
1	*E.cloacae*	VIM	10^4^	Negative
1	Influenza A			
25	Negative			Negative

*= Identification of significant pathogens was performed using MALDI TOF MS (Bruker Daltonics).

**Figure 2:**
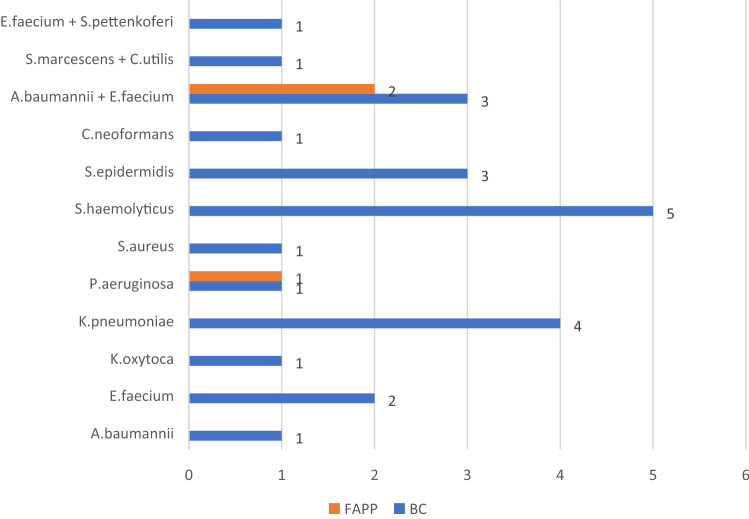
Pathogens detected in 24 positive Blood cultures. Blue bars indicate pathogens identified in positive blood cultures, while orange ones refer to pathogens both detected in BC and in respiratory specimens.

Additionally, positivity in blood cultures collected during admission was observed in 24 patients (24/152; 15.8%). Figure 2 shows the pathogens detected. In BAL or SPU, we detected the same microorganism only in two samples (shown by the orange bars in the graph).

## Discussion

Pneumonia is the leading cause of hospitalization and death worldwide, and this was the case even before the COVID-19 pandemic^26^. In cases of respiratory infection, it is important to quickly confirm or eliminate the involvement of pathogens (bacteria and/or viruses). This will not only improve the patient’s prognosis but will optimize the use of antimicrobials. Especially in the COVID era, we have come to understand the importance of the reasonable use of antibiotics to prevent unnecessary antibiotic treatments and curb the spread of antimicrobial resistance^22,28^. Getahun et al. also recommend reducing the test turnaround time in the diagnostic pathway of COVID-19 patients to curb the urge to use antibiotics, and they advocate that antimicrobial stewardship activities be included in the pandemic response^28^.

FAPP is an example of a rapid diagnostic tool that may de-escalate the use of initial empirical antibiotic therapy. In n our experience with FAPP, we observed that 61% of COVID-19 patients had coinfections. This finding is contrary to previous reports in which the positive rate is significantly higher^21,23,29^.

The most representative species in cases of coinfection was *S. aureus* (identified in 21 BAL samples and 27 SPU samples), especially MRSA (30/44; 62%), in which the *mec*A gene was detected; this was followed by gram-negative pathogens, which were detected in approximately 57% of the specimens (53/93). *P. aeruginosa* was the most common (21/93), followed by *K. pneumoniae* (15/93), *E. coli* (11/93), *E. cloacae* (10/93) and *A. baumannii* (8/93). Additionally, *H. influenzae*, which is generally considered difficult to obtain in cultures, was observed in twelve specimens^26,27^.

Compared with the FAPP findings, the results of the culture method were poor, but considering the higher sensitivity of the molecular method, this is to be expected. Additionally, although pathogens could be detected by both methods, their microbial counts were different. To comment on this obvious difference, it must be said that the FAPP reported bacteria semiquantitatively to the nearest whole log as genome copies/mL; therefore, comparisons of the microbial loads obtained by culture and FAPP are not completely reliable from a methodological point of view.

Our findings show that one or more pathogens were detected in 61% of the samples, which emphasizes that pneumonia and COVID-19 pneumonia are often polymicrobial infections^29–33^. On the other hand, the polymicrobial origins of pneumonia observed in this study confirm that adding antibiotics to treatment may be a prudent approach, especially when the clinical situation deteriorates. Considering the rapid results that FAPP provides and its useful information about the determinants of drug resistance in causative pathogens, it can truly be useful for guiding the empirical use of antibiotics toward more appropriate targeted therapy, particularly in patients admitted to the ICU, where the circulation of multidrug-resistant microorganisms is usually more worrisome^34–36^. Furthermore, FAPP allows the identification of the main respiratory pathogens and the detection of seven markers of resistance, and it has the potential to support clinical decision-making within a few hours of sampling. However, it must be said that the usefulness of the resistance markers detected by FAPP is strictly linked to the type of germs circulating in the setting in which it is used. In our case and in Italy in general, where the circulation of Enterobacterales KPC producers is high, this represents an important diagnostic plus^34–36^.

However, FAPP cannot completely replace traditional culture methods and antimicrobial susceptibility testing for two major reasons: the first is that FAPP cannot detect certain pathogens, such as members of Aspergillus spp., some enterobacteria, and some nonfermenting gram-negative bacilli, as shown by our findings; the second is based on the evidence that in addition to the resistance determinants detected by FAPP, many others can be expressed by pathogens, and therefore, traditional AST is still needed.

Finally, only 15.8% of the patients who underwent FAPP also had positive blood cultures. This finding is inconsistent with the data reported by Giacobbe et al.^20^. We can speculate that, especially during the first wave of COVID-19 in Italy, differences in geographic region can explain the observed data differences. Additionally, regarding BSIs, we are examining the different trends observed between patients in the first wave (whom the data used in this study represent) and those of patients in the second wave of COVID-19; in fact, in the latter wave, the incidence rate of BSIs rose by approximately 75% (data not presented), and these data are in line with what has already been described in the literature^20,34^.

In conclusion, rapid diagnostic systems, including more innovative and promising systems based on bionanotechnologies and nano-enabled biosensing that benefit from artificial intelligence, may represent the future of the diagnostic path for life-threatening infections, such as those caused by SARS CoV-2^37,38^.

## Data Availability

All data are provided in full in the results section of this paper.

## Abbreviations

FAPP: Biofire®FilmArray® Pneumonia panel plus
ICU: Intensive care unit
NICE: National Institute for Health and Care Excellence
BSI: Bloodstream infection
RDT: Rapid diagnostic test
BAL: Bronchoalveolar lavages
SPU: Sputum
ID: Microorganism identification
AST: Antimicrobial susceptibility testing
CTX-M: Bla _CTX-M_ gene
KPC: Bla _KPC_ gene
NDM: Bla _NDM_ gene
Oxa48-like: Bla _OXA48_ gene
VIM: Bla _VIM_ gene
IMP: Bla _IMP_gene
mecA/mecC: Methicillin resistance is conferred by the expression of the mecA/C geneS
MREJ: Right extremity junction region between the *mecA* cassette and the *orfX* gene
MIB: Micronaut panel
EUCAST: European committee on antimicrobial susceptibility testing
MALDI TOF MS: Matrix assisted laser desorption ionization time of flight
TTR: Time to results
